# The Treatment of Burns

**Published:** 1887-03

**Authors:** 


THE TREATMENT OF BURNS.—Altschul (*Monatsch. f. prakt. Dermat.*) reviews the treatment of burns, and gives the results of his own experience. Iodoform he regards as the application *par excellence* for burns of the second and third degrees; he prefers an iodoform gelatin of the strength of ten per cent., or, better still, an iodoform paste, of which the following is the formula:

| | | |
|---|---|---|
| ℞ | White wax, - - - - | ʒ ss |
| | Olive oil, - - - - | ℥ i |
| | Solution of subacetate of lead, - - | ʒ vi |
| | Iodoform, - - - - | ʒ ij. to iv |

—*American Med. Digest.*

---

BILLROTH writes the following antiseptics:

1. Iodoform is the safest and most effective of all manageable antiseptics.

2. Moss, wood, turf, mould and oakum are useful when there are discharges from the wound.

3. Corrosive sublimate in dilute solution is practically inert as an antiseptic to wounds, and renders the patient and surgeon alike liable to mercurial poisoning.

4. Carbolic acid, which is known to be dangerous in strong solutions, is, in very weak ones, as good for wound irrigation as clean water, but probably no better.—*Canada Lancet.*

